# Hydrogen Sulfide Protects against Chemical Hypoxia-Induced Injury by Inhibiting ROS-Activated ERK1/2 and p38MAPK Signaling Pathways in PC12 Cells

**DOI:** 10.1371/journal.pone.0025921

**Published:** 2011-10-05

**Authors:** Aiping Lan, Xinxue Liao, Liqiu Mo, Chuntao Yang, Zhanli Yang, Xiuyu Wang, Fen Hu, Peixi Chen, Jianqiang Feng, Dongdan Zheng, Liangcan Xiao

**Affiliations:** 1 Department of Physiology, Zhongshan School of Medicine, Sun Yat-sen University, Guangzhou, People's Republic of China; 2 Department of Cardiovasology, the First Affiliated Hospital, Sun Yat-sen University, Guangzhou, People's Republic of China; 3 Department of Anesthesiology, the First Affiliated Hospital, Sun Yat-sen University, Guangzhou, People's Republic of China; 4 Department of Cardiovasology, Region of Huang pu, the First Affiliated Hospital, Sun Yat-sen University, Guangzhou, People's Republic of China; Universidade Federal do Rio de Janeiro, Brazil

## Abstract

Hydrogen sulfide (H_2_S) has been proposed as a novel neuromodulator and neuroprotective agent. Cobalt chloride (CoCl_2_) is a well-known hypoxia mimetic agent. We have demonstrated that H_2_S protects against CoCl_2_-induced injuries in PC12 cells. However, whether the members of mitogen-activated protein kinases (MAPK), in particular, extracellular signal-regulated kinase1/2(ERK1/2) and p38MAPK are involved in the neuroprotection of H_2_S against chemical hypoxia-induced injuries of PC12 cells is not understood. We observed that CoCl_2_ induced expression of transcriptional factor hypoxia-inducible factor-1 alpha (HIF-1α), decreased cystathionine-β synthase (CBS, a synthase of H_2_S) expression, and increased generation of reactive oxygen species (ROS), leading to injuries of the cells, evidenced by decrease in cell viability, dissipation of mitochondrial membrane potential (MMP) , caspase-3 activation and apoptosis, which were attenuated by pretreatment with NaHS (a donor of H_2_S) or N-acetyl-L cystein (NAC), a ROS scavenger. CoCl_2_ rapidly activated ERK1/2, p38MAPK and C-Jun N-terminal kinase (JNK). Inhibition of ERK1/2 or p38MAPK or JNK with kinase inhibitors (U0126 or SB203580 or SP600125, respectively) or genetic silencing of ERK1/2 or p38MAPK by RNAi (Si-ERK1/2 or Si-p38MAPK) significantly prevented CoCl_2_-induced injuries. Pretreatment with NaHS or NAC inhibited not only CoCl_2_-induced ROS production, but also phosphorylation of ERK1/2 and p38MAPK. Thus, we demonstrated that a concurrent activation of ERK1/2, p38MAPK and JNK participates in CoCl_2_-induced injuries and that H_2_S protects PC12 cells against chemical hypoxia-induced injuries by inhibition of ROS-activated ERK1/2 and p38MAPK pathways. Our results suggest that inhibitors of ERK1/2, p38MAPK and JNK or antioxidants may be useful for preventing and treating hypoxia-induced neuronal injury.

## Introduction

Hydrogen sulfide (H_2_S) is a well- known cytotoxic gas. There is now increasing evidence that it is an endogenously produced gaseous messenger and, in particular, serves as a novel neuromodulator in the central nervous system (CNS) [1], [2]. H_2_S is usually stored as bound sulfane sulfur in neurons and astrocytes [3]. Upon neuron excitation or other stimuli, the bound sulfane sulfur then releases free H_2_S. A more recent study indicated that the estimated physiological concentration (free concentration) of H_2_S in the mice brain was around 14±3.0 nM [4] which is consistent with values reported by another group that tested H_2_S concentration using a novel method [3]. Physiological concentrations of H_2_S can potentiate the activity of the N-methyl-D-aspartate (NMDA) receptor and increase the induction of hippocampal long-term potentiation (LTP) [5], [6], which is associated with learning and memory. H_2_S can also induce calcium waves / elevation in both astrocytes and microglia [7], [8].

Importantly, accumulating evidence revealed that H_2_S may serve as an important neuroprotective agent. Kimura et al. firstly demonstrated that H_2_S protects primary rat cortical neurons from oxidative stress-induced injury [9]. H_2_S also protects cells against cytotoxicity caused by peroxynitrite, β-amyloid, hypochlorous acid and H_2_O_2_ [10], [11], [12], [13], [14]. Additionally, H_2_S attenuates lipopoly saccharide (LPS)-induced inflammation in microglia [15] and inhibits rotenone-induced apoptosis in human-derived dopaminergic neuroblastoma cell line (SH-SY5Y) [16]. We found recently that H_2_S protects PC12 cells against cobalt chloride (CoCl_2_, a chemical hypoxia mimetic agent)-induced injuries by enhancing heat shock protein 90 (HSP90) [17]. One of the key mechanisms underlying H_2_S neuroprotection is its antioxidation. H_2_S exerts its protective effect not only by enhancing reduced glutathione (GSH, a major cellular antioxidant) [9], [18], but also by scavenging reactive oxygen species (ROS) [11], [14], [17] and peroxynitrite [12] to suppress oxidative stress. In addition, H_2_S increases the redistribution of GSH into mitochondria, which also contribute to the neuroprotection from oxidative stress [18]. Another important H_2_S-triggered neuroprotective mechanism may be associated with its anti-inflammatory effect [15].

Recently, the roles of members of the mitogen-activated protein kinase (MAPK) family in H_2_S neuroprotection have attracted extensive attention. Mammals express at least three distinct groups of MAPKs, including extracellular signal-regulated protein kinase1/2 (ERK1/2), C-Jun N-terminal kinase (JNK) and p38MAPK. In neuronal cells, ERK1/2 is mainly activated by growth factor and is associated with cell proliferation, differentiation and development, whereas JNK and p38MAPK are preferentially activated by environmental stress and inflammatory cytokines, and have been shown to promote neuronal cell death [19], [20]. Hu et al. reported that H_2_S inhibits LPS-induced NO production in microglia via inhibition of p38MAPK [15] and that H_2_S protects SH-SY5Y cells against rotenone-induced apoptosis by inhibiting the p38/JNK signaling pathways [16]. In addition, H_2_S protects astrocytes against H_2_O_2_-induced neural injury via suppressing ERK1/2 activation [14]. These findings mentioned above suggest that the inhibition of ERK1/2 pathway or p38/JNK pathways may be involved in H_2_S neuroprotective effect in different cell models. However, whether both ERK1/2 and p38MAPK pathways participate in neuroprotection of H_2_S against chemical hypoxia-induced injury in PC12 cells is unclear.

Cobalt chloride (CoCl_2_) is a well-known hypoxia mimetic agent and can mimic the hypoxic response in many aspects [21]. CoCl_2_-mimicked hypoxia increases the level of HIF-1α protein [22], [23]. CoCl_2_ also functions as an oxidative stress-inducing factor since Co (II) can react with H_2_O_2_ by a Fenton-type reaction to produce ROS [24]. A recent study showed that H_2_O_2_ rapidly activates MAPKs, including ERK1/2, JNK and p38MAPK and that N-acetyl-L-cysteine (NAC), a free radical scavenger, dramatically inhibits H_2_O_2_-induced phosphorylation of ERK1/2, JNK and p38MAPK [25]. Furthermore, CoCl_2_ has been shown to activate p38MAPK in the perfused amphibian heart [26] and PC12 cells [27]. Since we have demonstrated that H_2_S protects PC12 cells against CoCl_2_-induced injury by inhibiting ROS overproduction, so, we speculated that H_2_S could prevent from CoCl_2_-induced injury via inhibition of ROS-activated ERK1/2 and p38MAPK pathways. This hypothesis is supported by the findings of present study that (1) CoCl_2_ elicited overproduction of ROS, and downregulated expression of CBS in PC12 cells; (2) CoCl_2_ upregulated expressions of both phosphorylated (p) ERK1/2 and p38MAPK; (3) NAC, an antioxidant and ROS scavenger, significantly depressed CoCl_2_-induced activation of both ERK1/2 and p38MAPK, and prevented CoCl_2_-induced injuries, including cytotoxicity, caspase-3 activation, apoptosis and loss of mitochondrial membrane potential(MMP); (4) U0126 (ERK1/2 inhibitor) or SB203580 (p38MAPK inhibitor) obviously attenuated CoCl_2_-induced injuries; (5) Si-ERK1/2 or Si-p38MAPK obviously attenuated CoCl_2_ induced cytotoxicity; (6) Similar to the effects of NAC, H_2_S protected PC12 cells against CoCl_2_-induced injuries, along with inhibition of CoCl_2_-induced ROS overproduction as well as activation of ERK1/2/p38MAPK. These findings provide a new insight into the mechanisms of H_2_S neuroprotection against chemical hypoxia-induced injury.

## Materials and Methods

### Materials

Sodium hydrosulfide, CoCl_2_, N-acetyl-L-cysteine (NAC), Hoechst33258, PI, RNase, dichlorofluorescein diacetate (DCFH-DA) and JC-1 were purchased from Sigma-Aldrich (St Louis, MO, USA). The Cell Counter Kit-8 (CCK-8)) was purchased from Dojindo Lab (Kumamoto, Japan). The DMEM medium and fetal bovine serum FBS) were supplied by Gibco BRL (Grand Island, NY, USA). Monoclonal anti-CBS antibody, anti-Cleaved-caspase-3 antibody, anti-p38 antibody, anti-p-p38 antibody, SP600125 and SB203580 were purchased from Cell Signaling Technology (Boston, MA, USA). anti-HIF1α,anti-p-ERK1/2 and anti-ERK1/2 antibody, anti-JNK antibody, anti-p-JNK antibody, were purchased from Bioworld company (Louis Park, MN, USA). U0126 (the inhibition of MEK1/2), anti-β-actin antibody, HRP-conjugated secondary antibody and BCA protein assay kit were purchased from Kangchen Bio-tech (Shanghai, China). Western Blot Detection Kit (ECL solution) was purchased from KeyGen Biotech (Nanjing, China).

### Cell culture and treatments

The rat pheochromocytoma cell line PC12 cells were purchased from Sun Yat-sen University Experimental Animal Center, and were grown in DMEM medium supplemented with 10% fetal bovine serum (FBS) at 37°C under an atmosphere of 5% CO_2_ and 95% air. According to our previous study [17], chemical hypoxia was achieved by adding CoCl_2_ at 600 µmol/L into the medium and cells were incubated in the presence of CoCl_2_ for the indicated times. The cytoprotective effects of H_2_S were observed by administering 400 µmol/L NaHS (a donor of H_2_S) for 30 min prior to exposure to CoCl_2_ for 24 h. In order to clarify the role of ERK1/2 or p38MAPK or JNK in CoCl_2_-induced injuries, cell were pretreated with U0126 (ERK1/2 inhibitor) for 120 min or SB203580 (p38MAPK inhibitor) for 60 min or SP600125 (JNK inhibitor) for 60min before exposure to CoCl_2_. NAC was administered 60 min prior to administration of 600 µmol/L CoCl_2_ for 24 h.

### Cell viability assay

PC12 cells were suspended in medium and plated at a density of 1×10^4^ cells/well in 96 well plates, and the cells viability was assessed by the Cell Counter Kit-8(CCK-8) assay. Cells were treated with 400 µmol/L NaHS for 30 min prior to administration of 600 µmol/L CoCl_2_ for 24 h. After the indicated treatments, 10 µl CCK-8 solution was added to each well of the plat and the cells in the plat were incubated for 4 h in the incubator. The absorbance at 450 nm was measured with a microplate reader(Molecular Devices , Sunnyvale, CA, USA). Means of 4 wells optical density (OD) in the indicated groups were used to calculated percentage of cells viability according to calculate percentage of cells viability according to the formula below:

Percentage of cells viability(%)  =  (OD treatment group / OD control group) ×100

Assuming that the absorbance of the control cells was 100%.The experiment was repeated 3 times.

### Nuclear Staining for assessment of apoptosis with Hoechst 33258

Apoptotic cell death was determined by using the Hoechst 33258 staining method. Cells were plated at a density of 1×10^6^ cells/well in 35 mm dishes. At the end of the indicated treatments, cells were harvested and fixed with 4% paraformaldehyde in 0.1 mol/L phosphate-buffered saline (PBS, pH 7.4) for 10 min. After rinsing with PBS, the nuclear DNA was stained with 5 mg/ml Hoechst 33258 dye for 10 min before being rinsed briefly with PBS and then visualized under a fluorescence microscope (Bx50-FLA; Olympus, Tokyo, Japan). Viable cells displayed a uniform blue fluorescence throughout the nucleus, whereas apoptotic cells showed condensed and fragmented nuclei.

### Flow cytometry (FCM) analysis of apoptosis

Treated PC12 cells were digested with trypsin (2.5 mg/ml), centrifuged at 350 g for 10 min and the supernatant was removed. Cells were washed twice with PBS and fixed with 70% ice-cold ethanol. Cells were then centrifuged at 350 g for 10 min, washed twice with PBS and adjusted to a concentration of 1×10^6^ cells/ml. Then, 0.5 ml RNase (1 mg/ml in PBS) was added to a 0.5 ml cell sample. After gentle mixing with PI (at a terminal concentration of 50 mg/L), mixed cells were filtered and incubated in the dark at 4°C for 30 min before flow cytometric analysis. The PI fluorescence of individual nuclei was measured by a flow cytometer (Beckman-Coulter, Los Angeles, CA, USA). (excitation: 488nm, emission: 615 nm). The research software matched with FCM was used to analyze all the data of DNA labeling. In the DNA histogram, the amplitude of the sub-G1 DNA peak, which is lower than the G1 DNA peak, represents the number of apoptotic cells. The experiment was repeated 3 times.

### Measurement of intracellular ROS generation

Intracellular ROS levels were determined by oxidative conversion of cell-Permeable DCFH-DA to fluorescent DCF. PC12 cells were cultured in 24 well plates. After the indicated treatments, cells were washed twice with PBS and 10 µmol/L DCFH-DA solution in serum-free medium was added and co-incubated for 30 min at 37°C. Cells were washed three times with PBS and DCF fluorescence was measured over the entire field of vision by using a fluorescent microscope connected to an imaging system (BX50-FLA; Olympus, Tokyo, Japan). Mean fluorescence intensity (MFI) from four random fields was analyzed by using IMAGEJ 1.41o software (National Institutes of Health (NIH), Bethesda, MD, USA). The MFI is used as an index of the amount of ROS. The experiment was repeated 3 times.

### Measurement of MMP

To determined the mitochondrial membrane potential the lipophilic cationic probe 5,5′,6,6′-tetrachloro-1,1′,3,3′-tetraethylbenzimidazol-carbocyanine iodide (JC-1) was used. In living cells, JC-1 exists either as a green fluorescent monomer at low membrane potential or as an orange-red fluorescent J-aggregate at high membrane potentials. The ratio of red/green JC-1 fluorescence is dependent on the mitochondrial membrane potential. In the present study, PC12 cells were cultured in 24 well plates and treated with 400 µmol/L NaHS for 30 min prior to administration of 600 µmol/L CoCl_2_ for 24 h. NAC was administered 60 min prior to administration of 600 µmol/L CoCl_2_ for 24 h. To evaluate MMP, JC-1 (5 mg/L) was added to cell cultures for 30 min at 37°C and fluorescence was measured over the entire field of vision using a fluorescent microscope connected to an imaging system (BX50-FLA; Olympus, Tokyo, Japan). The Ratios of red/green fluorescent densities from four random fields was analyzed by using IMAGEJ 1.41o software. The experiment was repeated 3 times.

### Western blot assay for expression of protein

After subjected to the indicated treatments, cells were harvested and lysed with cell lysis solution. Total protein in the cell lysate was quantified using the BCA protein assay kit. Sample buffer was added to cytosolic extracts, and after boiling for 5 min, equal amounts of supernatant from each sample were fractionated by 10% sodium dodecyl sulphate-polyacrylamide gel electrophoresis (SDS-PAGE). Total protein in the gel was transferred into polyvinylidene difluoride (PVDF) membranes. Membranes were blocked for 1.5 h at room temperature in fresh blocking buffer (0.1% Tween20 in Tris-buffered saline (TBS-T) containing 5% fat-free milk) and then incubated with either anti-CBS (1∶1000 dilution), anti-cleaved-caspase-3 (1∶1000), anti-p38 (1∶1000 dilution), anti-p-p38 (1∶1000 dilution), anti-HIF1α(1∶1000 dilution), anti-p-ERK1/2 (1∶1000 dilution), anti-ERK1/2 (1∶1000 dilution), anti-p-JNK, anti-JNK (1∶1000 dilution) or anti-β-actin antibodies (1∶5000 dilution) in freshly prepared TBS-T with 3% free-fat milk overnight with gentle agitation at 4°C. Following three washes with TBS-T, membranes were incubated with horseradish peroxidase-conjugated goat anti-rabbit secondary antibodies (1∶3000 dilution; Kangchen Biotech, shanghai, china) in TBS-T with 3% fat-free milk for 1.5 h at room temperature. Membranes were washed three times with TBS-T, developed in ECL solution (keygen Biotech, Nanjing, china) and visualized with X-ray film. Each experiment was repeated at least three times. For quantification, the film were scanned and analyzed by using IMAGEJ 1.41o software. And the density of specific bands was measured and normalized with the control band. The experiment was repeated 3 times.

### Gene knockdown

Small interfering RNA (Si-RNA) against rat p38MAPK and ERK1/2 subunit mRNA (NM-031020, NM-017347, NM-053842) was synthesized by GenePharma Co., Ltd (People's Republic of China). The Si-RNA of p38 and ERK1/2 (Si-p38 and Si-ERK1/2) and random non-coding RNA (Si-NC) were transfected into PC12 cells using Lipofectamine 2000, according to the manufacturer's instruction (Invitrogen, USA). Si-p38MAPK, Si-ERK1/2 and Si-NC (50 nmol/L) were incubated with the cells for 6 h in order to transfect into the cells. Efficiency of genetic silencing by Si-RNA was evaluated by western blot assay.

### Statistical analysis

All data are representative of experiments done in triplicate and are expressed as the mean ± SE. Differences between groups were analyzed by one-way analysis of variance(ANOVA) using SPSS 13.0 software, and followed by LSD post hoc comparison test. Statistical significance was defined as *P<*0.05.

## Results

### CoCl_2_ enhances expression of HIF-1α in PC12 cells

**Figure 1:**
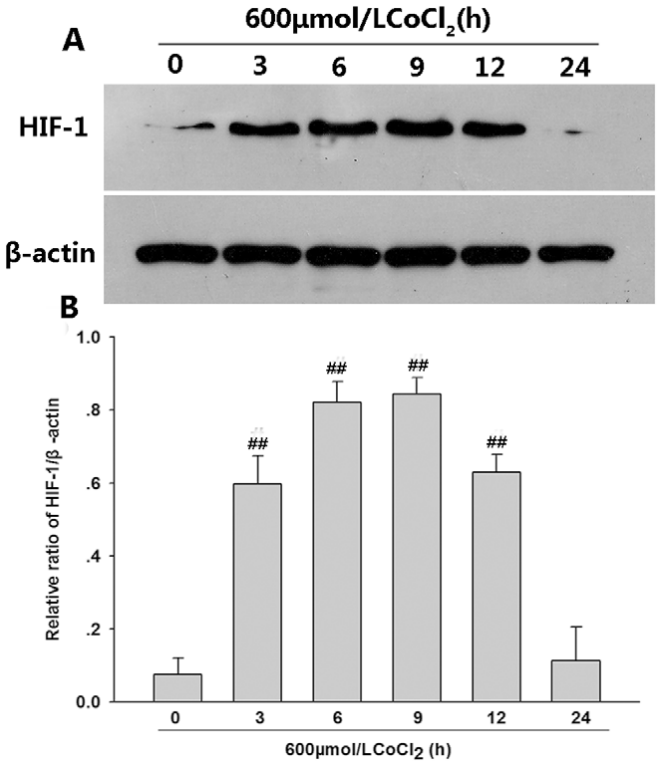
CoCl_2_-induced expression of HIF-1α in PC12 cells. (A) time course for the effects of CoCl_2_ on expression of HIF-1α detected by western blot analysis; (B) Denstiometric analysis for the results in (A) with the Image J 1.41o software. Data were shown as the mean ± SE (n = 3). ^##^ *P*<0.01 compared with control group (0h).

It is well known that CoCl_2_ is able to mimic transcriptional factor hypoxia-inducible factor-1 (HIF-1) activation by hypoxia which consists of two subunits: HIF-1α and HIF-1β. As shown in Figure 1, HIF-1α expression was lower in untreated PC12 cells (lane 1). However, its expression was significantly increased after 3 h exposure to 600 µmol/L CoCl_2_ and sustained up to 6<$>\vskip 1 \scale 80%\raster="rg1"<$>9 and 12 h, respectively. These results suggest that CoCl_2_ can mimic hypoxia in PC12 cells.

### CoCl_2_ inhibits expression of CBS in PC12 cells

#### Cystathionine-β-synthase

**Figure 2:**
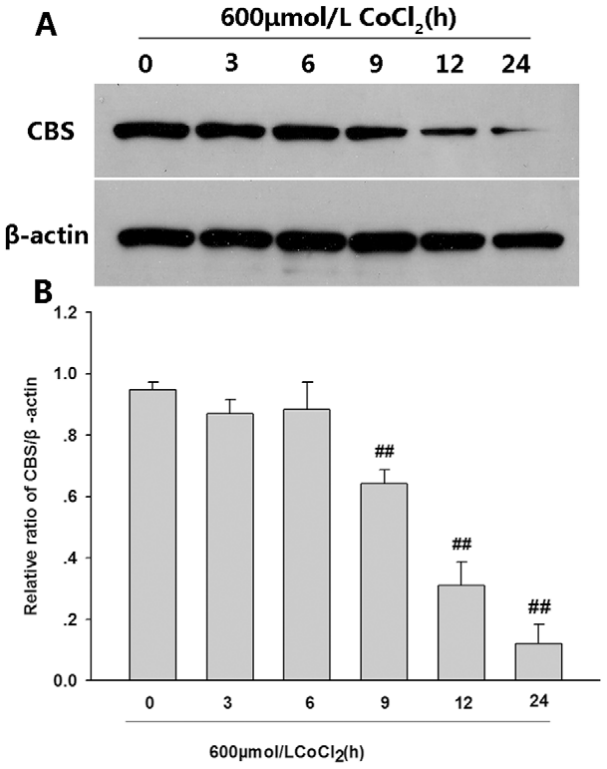
Effects of CoCl_2_ on the expression of CBS in PC12 cells. PC12 cells were treated with 600 µmol/L CoCl_2_ for different times. (A) The expression levels of CBS in PC12 cells were examined by western blot and β-actin was used as internal control. (B) Denstiometric analysis for the data from (A) with the Image J 1.41o software. Values are the mean ± SE (n = 3). ^##^ *P*<0.01 compared with control group (0h).

(CBS) is the major synthetic enzyme responsible for endogenous H_2_S generation in PC12 cells [28]. Western blot analysis was performed to evaluate whether CoCl_2_ decreases expression of CBS. As shown in Figure 2A and B, treatment with 600 µmol/L CoCl_2_ caused a significant down-regulation of CBS expression in PC12 cells at the indicated times (i.e. 9, 12 and 24 h after exposure to CoCl_2_). These data suggest that CoCl_2_ may decrease endogenous H_2_S production.

### H_2_S attenuates CoCl_2_-induced overproduction of ROS

**Figure 3:**
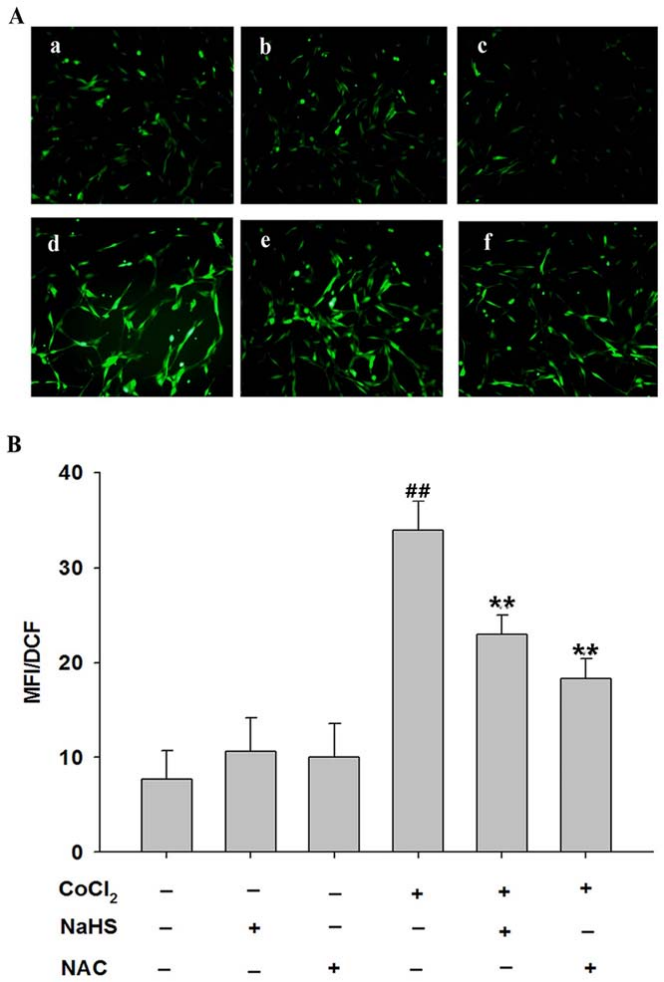
Effects of NaHS and NAC on CoCl_2_-induced overproduction of reactive oxygen species (ROS) in PC12 cells. (A) Random micrographs of dichlorofluorescein (DCF)-derived fluorescence in PC12 cells. A-a: Control, untreated cells; A-b: NaHS group, cells were treated with 400 µmol/L NaHS for 30 min alone; A-c: NAC group, cells were treated with 500 µmol/L NAC for 60 min alone; A-d: CoCl_2_-treated group, CoCl_2_ cells treated with 600 µmol/L CoCl_2_ for 6 h; A-e: NaHS+CoCl_2_ group, cells were preconditioned with 400 µmol/L NaHS for 30min prior to treatment with 600 µmol/L CoCl_2_ for 6 h; A-f: NAC+CoCl_2_ group, cells were preconditioned with 500 µmol/L NAC for 60 min prior to treatment with 600 µmol/L CoCl_2_ for 6 h; (B) Quantitative analysis of the mean fluorescence intensity in the indicated groups. Data are the mean ± SE (n = 3).^ ##^ *P*<0.01 compared with control, **P<0.01 compared with CoCl_2_-treated group.

As shown in Figure 3A-d and B, exposure of PC12 cells to 600 µmol/L CoCl_2_ for 6 h, significantly increased intracellular ROS levels. Pretreatment of PC12 cells with 400 µmol/L NaHS (a donor of H_2_S) for 30 min prior to exposure of cells to 600 µmol/L CoCl_2_ markedly reduced intracellular ROS levels (Figure 3A-e and B). To further demonstrate whether inhibition of H_2_S on CoCl_2_-induced ROS overproduction is associated with its antioxidation, NAC (a common ROS scavenger) was used. Similarly, pretreatment of PC12 cells with 500 µmol/L NAC for 60 min before exposure of cells to CoCl_2_ also obviously decreased intracellular ROS levels (Figure 3A-f and B). The results suggest that antioxidation of H_2_S may contribute to its inhibitory effect on CoCl_2_-induced generation of ROS.

### H_2_S inhibits CoCl_2_-induced phosphorylation of ERK1/2 and p38MAPK activated by ROS

**Figure 4:**
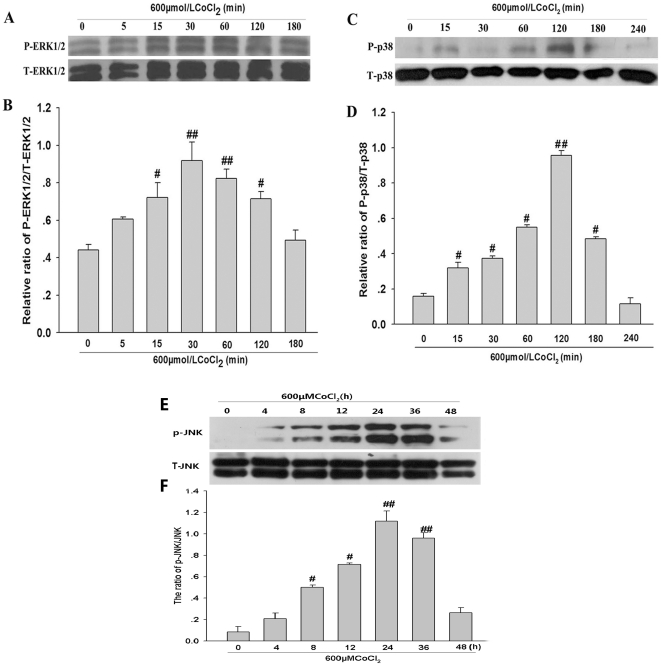
CoCl_2_-induced activation of ERK1/2, p38MAPK and JNK in PC12 cells. (A), (C) and (E) time course for the effects of CoCl_2_ on phosphorylation of ERK1/2, p38MAPK and JNK, respectively. (B), (D) and (F) Denstiometric analysis for the results in (A), (C) and (E), respectively. Data are presented as the mean ± SE (n = 3). ^#^ *P*<0.05, ^##^ *P*<0.01 compared to the control group (0min), respectively.

Findings of western blot analysis revealed that treatment of PC12 cells with 600 µmol/L CoCl_2_ induced expression of phosphorylated(p) ERK1/2 at specific times (i.e. 5, 15, 30, 60, 120, 180 min after exposure to CoCl_2_) , compare with control (Figure 4A and B). Within 15∼120 min after exposure to CoCl_2_, there was a sustained increase in expression of p-ERK1/2, which peaked at 30 min and 60 min (Figure 4A and B). However, CoCl_2_ treatment did not induce significant changes in expression of total ERK1/2 in the indicated times (Figure 4A and B). Similarly, as shown in Figure 4C and D, exposure of PC12 cells to CoCl_2_ also induced sustained expression of p-p38MAPK in the indicated times. The maximal expression of p-p38MAPK induced by CoCl_2_ appeared at 120 min. The expression of total p38MAPK was unchanged during exposure of cells to 600 µmol/L CoCl_2_ (Figure 4C). In addition, CoCl_2_ treatment also time-dependently increased expression of p-JNK in the indicated times (Figure 4E and F), but did not change expression of total JNK (Figure 4E).

**Figure 5:**
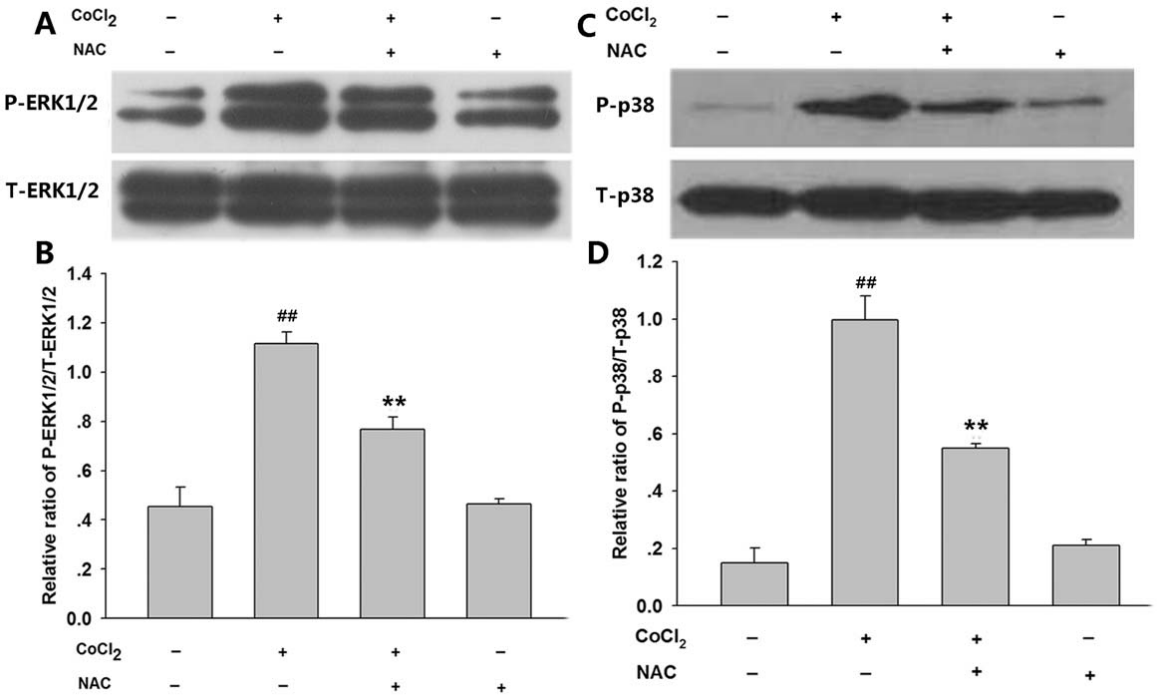
NAC attenuated CoCl_2_-induced ERK1/2 and p38MAPK phosphorylation in PC12 cells. PC12 cells were pretreated with 500 µmol/L NAC for 60 min prior to exposure of cells to 600 µmol/L CoCl_2_ for 30 min (A) and (B) or 120 min (C) and (D). Cell lysates were subjected to western blot analysis using anti-p-ERK1/2 and anti-ERK1/2 antibody (A) and (B) or anti-p-p38 and anti-p38 antibody (C) and (D). (B) and (D) show denstiometric analysis for the data from (A) or (C), respectively. Data are presented as mean ± SE from independent experiments preformed in triplicate. ^##^ *P*<0.01 compared to the control group, **P<0.01 compared to the CoCl_2_ group.

We also explored roles of ROS in CoCl_2_-induced expressions of p-ERK1/2 and p-p38MAPK. As shown in Figure 5A and B, pretreatment of PC12 cells with 500 µmol/L NAC for 60 min prior to exposure of cells to CoCl_2_ at 600 µmol/L markedly suppressed overexpression of p-ERK1/2 induced by CoCl_2_ treatment for 30 min. NAC alone did not change expression of p-ERK1/2. In addition, NAC pretreatment also exerted similar inhibitory effect on CoCl_2_-induced overexpression of p-p38MAPK (Figure 5C and D). The above findings suggest that CoCl_2_-induced phosphorylation of ERK1/2 and p38MAPK is triggered by ROS.

**Figure 6:**
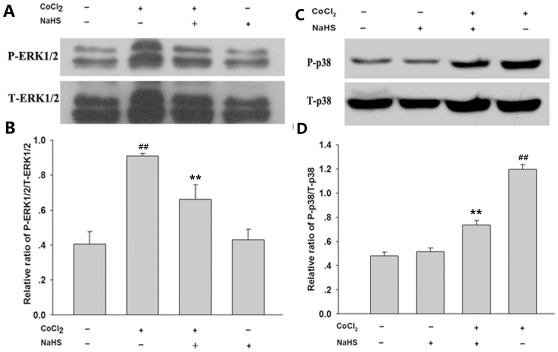
NaHS attenuated CoCl_2_-induced ERK1/2 and p38 MAPK phosphorylation in PC12 cells. PC12 cells were preconditioned with 400 µmol/L NaHS for 30min before exposure of cells to 600 µmol/L CoCl_2_ for 30 min (A) and (B) or for 120min (C) and (D). Cell lysates were subjected to western blot analysis using anti-p-ERK1/2 and anti-ERK antibody (A and B) or anti-p-p38 and anti-p38 antibody (C) and (D). Panels (B) and (D) show denstiometric analysis for the data from (A) or (C), respectively. Data are presented as mean ± SE from independent experiments preformed in triplicate. ^##^ *P*<0.01 compared to the control group, **P<0.01 compared to the CoCl_2_ group.

Importantly, we observed that H_2_S can depress ROS-activated ERK1/2 and p38MAPK induced by CoCl_2_. As shown in Figure 6A and B, exposure of PC12 cells to 600 µmol/L CoCl_2_ for 30 min obviously upregulated expression of p-ERK1/2, this effect was markedly suppressed by pretreatment of cells with 400 µmol/L NaHS for 30 min before exposure to CoCl_2_ (Figure 6A and B). Additionally, treatment of PC12 cells with 600 µmol/L CoCl_2_ for 120 min also enhanced expression of p-p38MAPK, which was attenuated by pretreatment of cells with 400 µmol/L NaHS for 30 min prior to CoCl_2_ treatment (Figure 6C and D). However, pretreatment with NaHS did not alter the increased expression of JNK induced by CoCl_2_ exposure (data not shown). NaHS at 400 µmol/L alone did not affect the basal expression of p-ERK1/2 and p-p38MAPK (Figure 6).

### MAPK pathway mediates CoCl_2_-induced injuries in PC12 cells

**Figure 7:**
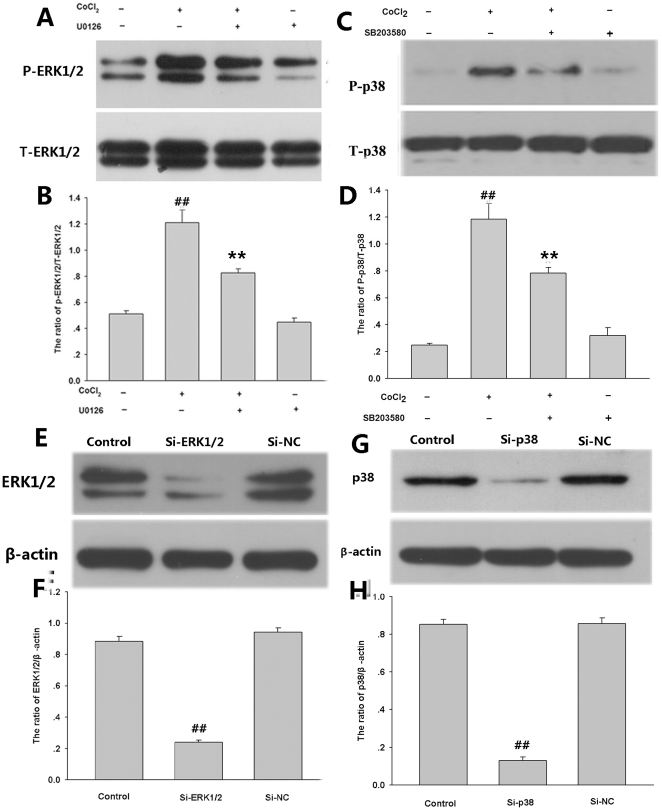
Effects of kinase inhibitors on CoCl_2_-induced phosphorylation of p38MAPK and ERK1/2 as well as the gene silencing effect on their expressions. PC12 cells were preconditioned with 10 µmol/L MEK1/2 (upstream of ERK1/2) inhibitor U0126 for 120 min and 20 µmol/L p38MAPK inhibitor SB203580 for 60 min before exposure of cells to 600 µmol/L CoCl_2_ for 30 min (A, B) and 120 min (C, D), respectively. Panels (B) and (D) show denstiometric analysis for the data from (A) or (C), respectively. (E) and (G) PC12 cells were co-cultured with small interfering RNA (Si-ERK1/2 and Si-p38MAPK) or random non-coding RNA (Si-NC) at 50 nmol/L for 6 h. Expressions of ERK1/2 and p38MAPK were detected by Western blot assay. Panels (F) and (H) show denstiometric analysis for the data from (E) and (G), respectively. Data are presented as mean ± SE from independent experiments preformed in triplicate. ^##^ *P*<0.01 compared to the control group, **P<0.01 compared to the CoCl_2_ group.

To dissect the roles of ERK1/2, p38MAPK and JNK in CoCl_2_-induced injuries, we firstly tested effects the kinases inhibitor on CoCl_2_-induced exoressions of p-ERK1/2 and p-p38MAPK. PC12 cells were pretreated with MEK1/2(upstream of ERK1/2) inhibitor U0126 or SB203580 (p38MAPK inhibitor), respectively, then followed by exposure of cells to 600 µmol/L CoCl_2_. As shown in Figure 7A and C, pretreatment of with ERK1/2 inhibitor U0126 (10 µmol/L) for 120 min or p38MAPK inhibitor SB203580 (20 µmol/L) for 60 min, blocked CoCl_2_-induced phosphorylation of ERK1/2 or p38MAPK, respectively. Additionally, gene silencing experiments (Figure 7E and G) showed that genetic silencing of ERK1/2 or p38MAPK by RNAi (Si-ERK1/2 or Si-p38MAPK) attenuated expression of ERK1/2 or p38MAPK, respectively.

**Figure 8:**
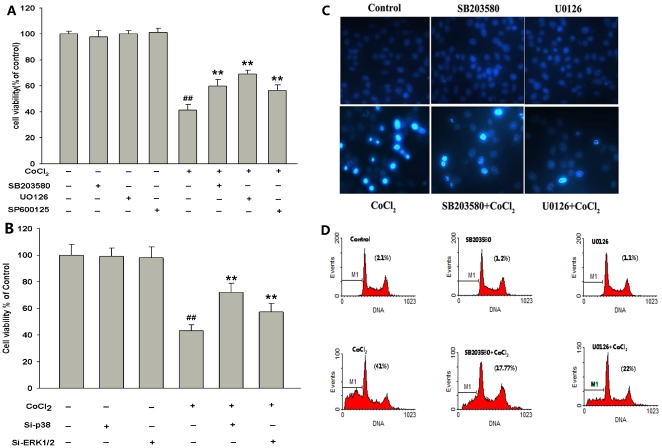
ERK1/2, p38MAPK and JNK pathways mediated CoCl_2_-induced injuries in PC12 cells. (A) and (B) The cell viability was assessed by the CCK-8 reduction method described in ‘Materials and methods’. The cells were treated with the indicated treatments. (C) Morphological changes in apoptotic cells assessed by Hoechst 33258 staining. Arrow indicates cells with apoptotic nuclear condensation and fragmentation. Control: untreated cells; SB203580: cells were treated with 20 µmol/L SB203580 for 60 min alone; UO126: cells were treated with 10 µmol/L UO126 for 120min alone; CoCl_2_: cells were treated with 600 µmol/L CoCl_2_ for 48 h; SB203580+ CoCl_2_: cells were pretreated with p38 inhibitor SB203580 (20 µmol/L) for 60 min followed by exposure of cells to 600 µmol/L CoCl_2_ for 48 h; UO126+ CoCl_2_: cells were pretreated with ERK1/2 inhibitor UO126 (10 µmol/L) for 120 min followed by exposure of cells to 600 µmol/L CoCl_2_ for 48 h. (D) Results from FCM analysis. Data are the mean ± SE (n = 3).^ ##^ *P*<0.01 compared with control; **P<0.01 compared with CoCl_2_-treated group.

Next, we examined the roles of ERK1/2, p38MAPK and JNK pathways in CoCl_2_-induced cell injuries. As shown in Figure 8A and B, after PC12 cells were treated with CoCl_2_ at 600 µmol/L for 24 h, the cell viability was dramatically reduced to (41.28±4.44)% (*P*<0.01) compared with control group. However, when cells were preconditioned with 20 µmol/L SB203580 for 60 min or 10 µmol/L U0126 for 120 min or 10 µmol/L SP600125 (inhibitor of JNK) for 60 min, followed by exposure to 600 µmol/L CoCl_2_ for 24 h, the cell viability was considerably enhanced, respectively (Figure 8A). In addition, co-incubation of cells with Si-p38 or Si-ERK1/2 for 6 h also blocked CoCl_2_-induced inhibitory effect on cell viability (Figure 8B). These data indicate that ERK1/2, p38MAPK and JNK pathways are involved in CoCl_2_-induced cytotoxicity.

We further examined whether ERK1/2 and p38MAPK pathways participate in CoCl_2_-induced apoptosis. Our findings showed that the cells, treated with 600 µmol/L CoCl_2_ for 48 h appeared typical characteristics of apoptosis, including the condensation of chromatin, the shrinkage of nuclear and a few of apoptotic bodies (Figure 8C). However, preconditioning of cells with 10 µmol/L U0126 for 120 min prior to CoCl_2_ treatment obviously reduced the number of cells with nuclear condensation and fragmentation (Figure 8C). Pretreatment of cells with 20 µmol/L SB203580 for 60 min before exposure to CoCl_2_, also inhibited CoCl_2_-induced apoptosis (Figure 8C). Alone, U0126 (10 µmol/L) or SB203580 (20 µmol/L) did not significantly alter morphology or apoptotic percentage of PC12 cells compared with the control (Figure 8C). In addition, the data from FCM analysis further demonstrated that exposure of cells to 600 µmol/L CoCl_2_ for 48 h increased the percentage of apoptotic PC12 cells (Figure 8D). However, the apoptotic effect of CoCl_2_ treatment was reversed by pretreatment of cells with U0126 or SB203580, respectively (Figure 8D).

**Figure 9:**
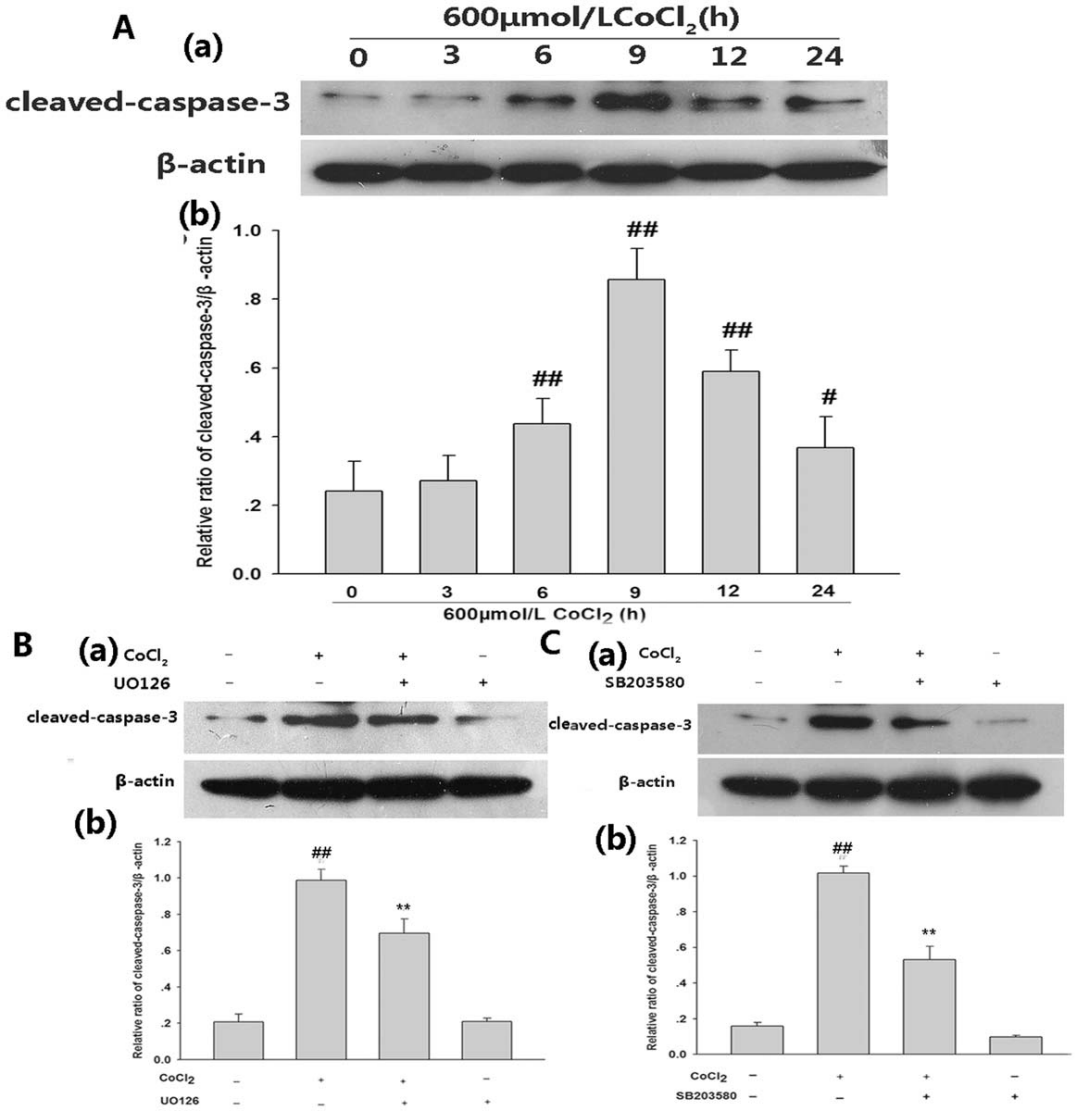
Kinase inhibitors suppressed CoCl_2_-induced expression of cleaved-caspase-3. (A) time course for the effects of CoCl_2_ on expression of cleaved-casepase-3. (A-b) Denstiometric analysis for the results in (A-a). Data are presented as the mean ± SD from independent experiments performed in triplicate. ^#^ *P*<0.05,^##^ *P*<0.01 compared to the control group. (B) and (C) PC12 cells were preconditioned with 10 µmol/L MEK1/2 (upstream of ERK1/2) inhibitor U0126 for 120 min (B) and 20 µmol/L p38MAPK inhibitor SB203580 for 60 min (C) before exposure of cells to 600 µmol/L CoCl_2_ for 9 h. Panels B-b and C-b show denstiometric analysis for the data from B-a or C-a, respectively. Data are presented as mean ± SE from independent experiments preformed in triplicate. ^##^ *P*<0.01 compared to the control group, **P<0.01 compared to the CoCl_2_ group.

Furthermore, we examined the roles of ERK1/2 and p38MAPK pathways in CoCl_2_-induced caspase-3 (apoptotic effector). The results of western blot analysis showed that exposure of cells to 600 µmol/L CoCl_2_ enhanced the expression of cleaved caspase-3 within 6 to 24 h(Figure 9A-a,b). Pretreatment of cells with 10 µmol/L U0126 or 20 µmol/L SB203580 prior to CoCl_2_ treatment inhibited CoCl_2_-induced expression of cleaved-caspase-3, respectively (Figure 9B-a, b and C-a, b). These results further indicated that both ERK1/2 and p38 MAPK pathways play important roles in CoCl_2_-induced apoptosis of PC12 cells.

### H_2_S and NAC protect PC12 cells against CoCl_2_-induced injuries

**Figure 10:**
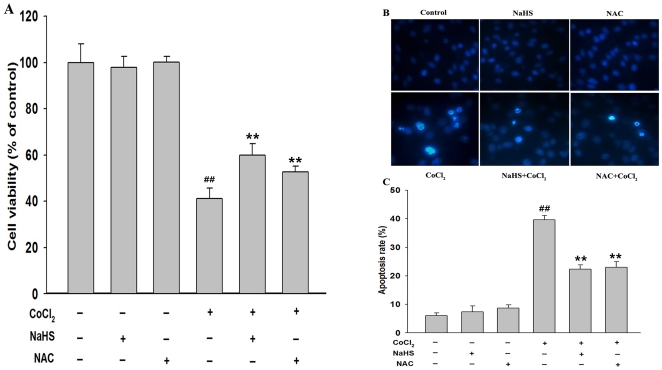
H_2_S and NAC protected PC12 cells against CoCl_2_-induced injuries. (A) The cell viability was assessed by the cell counter kit (CCK-8) described in ‘Materials and methods’. PC12 cells were treated with the indicated treatments. (B) Morphological changes in apoptotic cells assessed by Hochest 33258 staining. Arrow indicated cells with apoptotic nuclear condensation and fragmentation. Control: untreated cells; NaHS: cells were treated with 400 µmol/L NaHS for 30 min alone; NAC: cells were treated with 500 µmol/L NAC for 60 min alone; CoCl2: cells were treated with 600 µmol/L CoCl_2_ for 48 h; NaHS+CoCl_2_: cells were preconditioned with 400 µmol/L NaHS for 30 min prior to treatment with 600 µmol/L CoCl_2_ for 48 h; NAC+CoCl_2_: cells were preconditioned with 500 µmol/L NAC for 60 min prior to treatment with 600 µmol/L CoCl_2_ for 48 h; (C) The apoptotic rate was analysed with a cell counter of Image J 1.41o software. Data are the mean ± SE (n = 3). ^##^ *P*<0.01 compared to the control group, **P<0.01 compared to the CoCl_2_ group.

Protective effects of H_2_S and NAC were examined on CoCl_2_-induced cytotoxicity and apoptosis in PC12 cells. As shown in Figure 10A, when PC12 cells were exposed to 600 µmol/L CoCl_2_ for 24 h, the cell viability was reduced to (41.28±4.14)% compared with control group (*P*<0.01). Pretreatment of cells with 400 µmol/L NaHS for 30 min prior to exposure to CoCl_2_ significantly increased cell viability to (59.83±5.0)% (*P*<0.01) compared to the CoCl_2_-treated group, indicating that H_2_S suppresses CoCl_2_-induced cytotoxicity. Pretreatment with 500 µmol/L NAC for 60 min had similar cytoprotective effect against CoCl_2_-induced cytotoxicity (Figure 10A).

**Figure 11:**
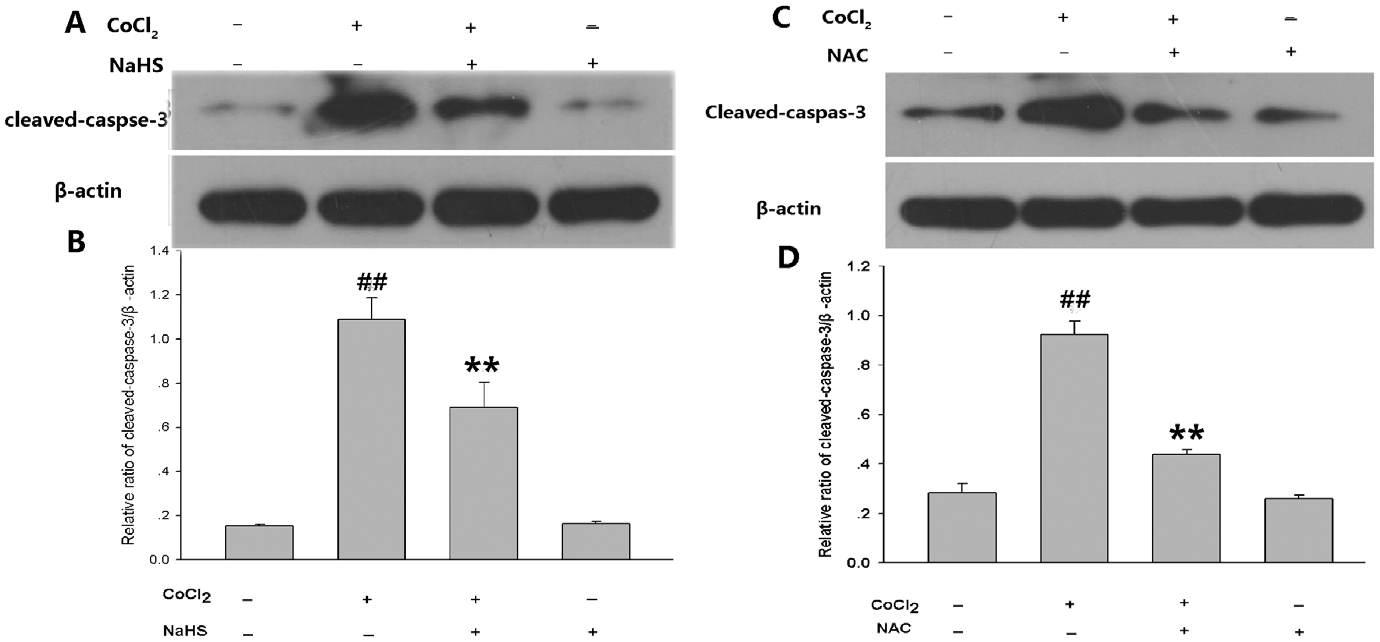
H_2_S and NAC suppressed CoCl_2_-induced expression of cleaved-caspase-3. (A) PC12 cells were preconditioned with 400 µmol/L NaHS for 30 min before exposure of cells to 600 µmol/L CoCl_2_ for 9 h; (B) Denstiometric analysis for the results in (A). (C) PC12 cells were preconditioned with 500 µmol/L NAC for 60 min before exposure of cells to 600 µmol/L CoCl_2_ for 9 h. (D) Denstiometric analysis for the results in (C). Data are presented as the mean ± SE from independent experiments performed in triplicate. ^##^ *P*<0.01 compared to the control group, ^**^ *P*<0.01 compared to the CoCl_2_ group.

We also observed cytoprotection of H_2_S and NAC against CoCl_2_-induced apoptosis in PC12 cells. As shown in Figure 10B and C, Pretreatment of PC12 cells with 400 µmol/L NaHS for 30 min or 500 µmol/L NAC for 60 min before exposure to 600 µmol/L CoCl_2_ for 48 h significantly attenuated CoCl_2_-induced apoptosis, respectively. In addition, we examined the effects of NaHS and NAC on the expression of cleaved-caspase-3 induced by CoCl_2_ treatment in PC12 cells, our findings demonstrated that, pretreatment with NaHS (Figure 11A and B) and NAC (Figure 11C and D) blocked CoCl_2_-induced the expression of cleaved-caspase-3 in PC12 cells .

**Figure 12:**
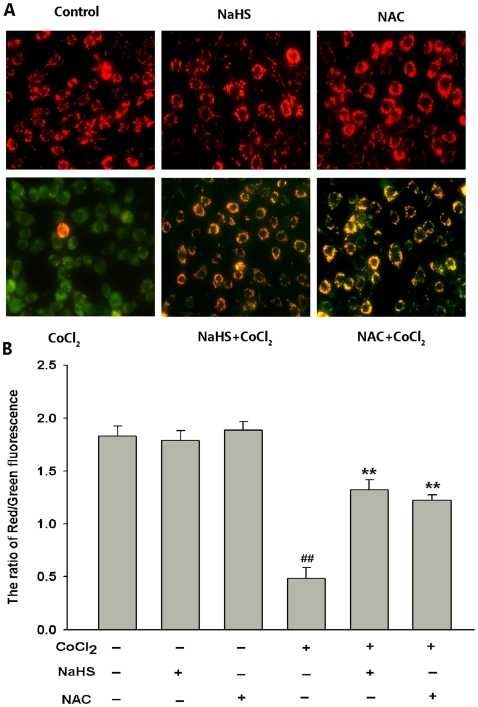
H_2_S and NAC protected PC12 cells against CoCl_2_-induced mitochondrial insult. MMP was assessed by JC-1 staining. Dual emission images (527 and 590nm) represent the signals from monomeric (green) and J-aggregate (red) JC-1 fluorescence in PC12 cells. (A) Control, untreated cells; NaHS, cells were treated with 400 µmol/L NaHS for 30 min alone; NAC, cells were treated with 500 µmol/L NAC for 60 min alone; CoCl_2_, cells were treated with 600 µmol/L CoCl_2_ for 24 h; NaHS+ CoCl_2_, cells were preconditioned with 400 µmol/L NaHS for 30 min prior to treatment with 600 µmol/L CoCl_2_ for 24 h; NAC+CoCl_2_, cells were preconditioned with 500 µmol/L NAC for 60 min prior to treatment with 600 µmol/L CoCl_2_ for 24 h; (B) Quantitative analysis of the ratio of Red/Green fluorescence in each group, by using Image 1.41o software. Data are the mean ± SE (n = 3).^ ##^ *P*<0.01 compared to the control group; **P<0.01 compared to the CoCl_2_ group.

Furthermore, our findings showed that both H_2_S and NAC can protect PC12 cells against CoCl_2_-induced mitochondrial insult. As shown in Figure 12A and B, when PC12 cells were treated with 600 µmol/L CoCl_2_ for 24 h, the MMP was dramatically reduced 0.3 fold, as shown by a decrease in MFI, compared with control cells (*P*<0.01). However, preconditioning with 400 µmol/L NaHS for 30 min or 500 µmol/L NAC for 60 min prior to CoCl_2_ treatment for 24 h obviously attenuated CoCl_2_-induced dissipation of MMP, increasing 2.8 fold or 2.7 fold of MMP compared with the one in CoCl_2_-treated group (*P*<0.01), respectively. NaHS (400 µmol/L) or NAC (500 µmol/L) alone did not measurably affect MMP.

## Discussion

It is well documented that hypoxia/ischemia is one of the main causes of secondary neuronal injury because this condition results in the production of ROS which can attack nucleic acids, proteins and membrane phospholipids [29], [30]. Thus, it is very important to explore the mechanisms underlying hypoxia/ischemia-induced neuronal injury or neuroprotective effects in various cell types or cell models. CoCl_2_-induced cell death in PC12 cells may serve as a simple and convenient in vitro model of hypoxia-induced neuronal injury to elucidate the mechanisms responsible for hypoxia-linked cell death and search its treatment methods because CoCl_2_ can mimic hypoxic/ischemic condition including ROS production, loss of MMP, etc. in neuronal cells [17], [21], [24], [27], [29]. In this study, we observed that CoCl_2_ treatment induced expression of HIF-1α, which is enhanced under hypoxic conditions, confirming that CoCl_2_ can mimic hypoxia in PC12 cells. Our results are consistent with the ones reported by Wang, et al. [29]. Recently, we have investigated the cytoprotection of H_2_S against chemical hypoxia-induced injury in this experimental model. We found that HSP90 mediates neuroprotection of H_2_S against CoCl_2_-induced insult [17]. Based on our previous study, this study was designed to further explore the molecular mechanisms of H_2_S neuroprotective effect, in particular, focusing on that (1) whether CoCl_2_-induced ROS activates ERK1/2, p38MAPK and JNK pathway? If so, (2) whether ROS-activated ERK1/2 and p38MAPK pathways participate in neuroprotection of H_2_S against CoCl_2_-induced injury in PC12 cells?

To investigate whether ROS is involved in CoCl_2_-induced injury, PC12 cells were pretreated with NAC (a ROS scavenger) prior to exposure of cells to CoCl_2_. We found that CoCl_2_ induced not only ROS production, but also initiated injuries of PC12 cells, including decrease in cell viability, loss of MMP and caspase-3 activation, as well as an increase in the number of apoptotic cells. These cell injuries were significantly prevented by NAC pretreatment, indicating that CoCl_2_-induced neuronal injuries are due to its induction of ROS. Our findings are comparable with the recent evidence that NAC scavenges H_2_O_2_-induced ROS production and inhibits apoptosis of PC12 cells induced by H_2_O_2_ [25]. Interestingly, we observed that NaHS (a donor of H_2_S) shared similar neuroprotective properties with NAC with a comparable potency in this experimental model. This may be supported by the ability of H_2_S in (1) inhibiting hypochlorous acid-mediated oxidative damage [13]; and (2) inhibiting peroxynitrite-mediated protein nitration and cytotoxicity [12]; (3) inhibiting generation of ROS induced by CoCl_2_ [17]. Additionally, H_2_S readily scavenges H_2_O_2_, an important source of oxidative stress in most cells in vitro [31] and increases the production of reduced GSH [9].

Accumulating evidence indicated that members of MAPK family may play a critical role in neuronal apoptosis [25], [32], [33], [34], [35]. Liu et al. reported that hypoxia and reoxygenation-induced apoptosis is associated with p38MAPK activity in culture rat cerebellar granule neurons [34]. On the other hand, members of MAPK are activated by ROS generated intracellularly, as well as by H_2_O_2_ administered [25], [36], [37]. Hypoxia also leads to p38MAPK activation [27], [34], [38]. Based on the above previous studies, we explore influence of CoCl_2_ on phosphorylation of ERK1/2, p38MAPK and JNK in PC12 cells. The results of present study showed that exposure of PC12 cells to CoCl_2_ significantly upregulated expressions of p-ERK1/2, p-p38MAPK and p-JNK. Zou et al. also observed that p38MAPK is markedly activated in CoCl_2_-treated PC12 cells, but did not test the changes in both ERK1/2 and JNK activation [27]. Our findings extend understanding of effect of CoCl_2_ on MAPK pathways in PC12 cells.

Notably, our study further demonstrated that MEK1/2 (upstream of ERK1/2) inhibitor U0126 or p38MAPK inhibitor SB203580 or JNK inhibitor SP600125 dramatically abolished CoCl_2_-induced injuries, evidenced by an increase in cell viability and decreases in caspase-3 activation, apoptotic cells, ROS production and MMP loss (data not shown). Similarly, genetic silencing of ERK1/2 or p38MAPK by RNAi (Si-ERK1/2 or Si-p38MAPK) also inhibited CoCl_2_-induced cell injury. These data suggest that ERK1/2, p38MAPK and JNK pathways mediate CoCl_2_-induced injuries. Our findings are consistent with those of the previous studies [27], [34] and comparable with the recent evidence that the members of MAPKs, including ERK1/2, JNK and p38MAPK mediate H_2_O_2_-induced neuronal apoptosis [25]. In addition, our results are supported by other previous studies [39], [40]. A MEK inhibitor has been shown to protect against damage resulting from focal cerebral ischemia [39], and H_2_O_2_-induced apoptosis is mediated by ERK1/2 phosphorylation in mouse fibroblast cells [40]. However, the findings of this study contradict the assertion by Xia et al. [41] and counter the idea that MEK/ERK signaling plays a critical role in cell survival [42]. Taken together, ERK1/2 being a protective signal and JNK/p38MAPKs being a proapoptotic signal do not always hold true and may depend on the nature of the death stimulus, the cell type, the duration of activation, and probably, most importantly, the activities of other signaling pathways [42], [43].

Since we found that ROS was involved in CoCl_2_-induced cell injuries, we further dissect whether CoCl_2_ activation of ERK1/2 and p38MAPK is due to its induction of ROS. It was shown that pretreatment of PC12 cells with NAC (a ROS scavenger) significantly attenuated CoCl_2_-induced phosphorylation of ERK1/2 and p38MAPK. Collectively, the above results of present study support the notion that CoCl_2_ induction of ROS activates ERK1/2 and p38MAPK pathways which mediates CoCl_2_-induced injuries in PC12 cells. Our findings are supported by the previous studies [25], [33], [34].

Importantly, we found that pretreatment of PC12 cells with NaHS inhibited not only CoCl_2_-induced ROS production, but also expressions of both p-ERK1/2 and p38MAPK induced by CoCl_2_, suggesting that H_2_S suppresses ROS-activated ERK1/2 and p38MAPK pathways, which may be one of important mechanisms underlying the neuroprotection of H_2_S against chemical hypoxia-induced neuronal injury. However, we did not find the inhibitory effect of NaHS on CoCl_2_-induced expression of JNK (data not shown). The involvement of p38MAPK in the cytoprotective effect of H_2_S has also been reported by other groups. Rinaldi et al. indicated that H_2_S prevents apoptosis of human polymorphonuclear cells via inhibition of p38MAPK and caspase-3 [44]. Hu et al. recently also reported that H_2_S suppresses LPS-inflammation by inhibition of p38MAPK in microglia [15] and that H_2_S protects SH-SY5Y cells against rotenone-induced apoptosis via suppression of p38 and JNK MAPK activation [16]. In addition, the stimulatory effect of H_2_S on glutamate uptake which can increase GSH production may be associated with the inhibition of ERK MAPK signaling pathway [14]. Overall, the above findings suggest that inhibition of ERK1/2 and p38MAPK may play a critical role in the cytoprotective effects of H_2_S.

In conclusion, the present study reveals that a concurrent activation of ERK1/2, p38MAPK and JNK pathways is involved in CoCl_2_-induced neuronal injuries and that H_2_S protects PC12 cells against chemical hypoxia-induced injuries via inhibition of ROS-activated ERK1/2 and p38MAPK pathways. Continued attempts to identify novel target molecules of ERK1/2 and p38MAPK activation and to clarify their cross-talk with upstream and downstream signaling molecules will pave the way for understanding of cellular and molecular regulatory mechanisms of H_2_S neuroprotection.
